# Self‐Efficacy, Self‐Care, Glycaemic Control, and Quality of Life Among Adults With Type 2 Diabetes: A Scoping Review

**DOI:** 10.1002/edm2.70241

**Published:** 2026-05-20

**Authors:** Omar Alqaisi, Faten Harb, Safia Darwish, Mohammed Dibas, Suzan Gharib, Patricia Tai

**Affiliations:** ^1^ Nursing Department Al‐Zaytoonah University Amman Jordan; ^2^ Department of Allied Medical Professions ‐ Associated Nursing Program Technical College‐Middle East University Amman Jordan; ^3^ Nursing Administration Department, Faculty of Nursing Menoufia University Menoufia Egypt; ^4^ Department of Medicine, Faculty of Medicine and Health Sciences An‐Najah National University Nablus Palestine; ^5^ Nursing Department Hashemite University Zarqa Jordan; ^6^ Department of Oncology University of Saskatchewan, 105 Administration Place Saskatoon Saskatchewan Canada

**Keywords:** glycaemic control, HbA1c, health literacy, health‐related quality of life, self‐care behaviours, self‐efficacy, type 2 diabetes mellitus

## Abstract

**Introduction:**

Type 2 diabetes mellitus (T2DM) is no longer just a metabolic diagnosis; it is a daily test of confidence, endurance and quality of life. Traditional measures such as haemoglobin A1c (HbA1c) provide only a partial picture, while patients' beliefs about their ability to manage diabetes may be equally crucial. This scoping review examines how self‐efficacy and self‐care behaviours intersect with glycaemic control and health‐related quality of life (HRQoL) in adults with T2DM across diverse health systems and cultures.

**Methods:**

Following the Arksey and O'Malley framework and PRISMA‐ScR guidelines, a systematic search was conducted across four databases covering January 2016 to March 2026. Studies were eligible if they included adults aged ≥ 18 years with confirmed T2DM and examined at least one of the following: diabetes self‐efficacy, self‐care behaviours, glycaemic control (HbA1c) or HRQoL. Thirteen studies encompassing 5784 adults with T2DM were included, spanning cross‐sectional, longitudinal, quasi‐experimental and randomised controlled trial designs across seven countries.

**Results:**

Diabetes management self‐efficacy consistently outperformed knowledge in predicting self‐care behaviours and HbA1c, with patients holding stronger efficacy beliefs demonstrating better metabolic control and higher HRQoL. Interventions grounded in social cognitive theory and empowerment approaches achieved clinically meaningful HbA1c reductions alongside improvements in self‐care and quality of life. Advanced modelling identified a psychological cascade whereby health literacy enhanced empowerment and self‐efficacy, which in turn drove self‐care and improved glycaemic and HRQoL outcomes. Cluster analyses further revealed distinct high‐risk subgroups, underscoring the need for stratified, individualised interventions.

**Conclusion:**

In T2DM, self‐efficacy consistently emerged as a central psychological factor associated with self‐care behaviour, glycaemic control and quality of life. The available evidence suggests that theoretically grounded, individually tailored interventions addressing psychological and behavioural determinants, including self‐efficacy, self‐care, empowerment and resilience, hold promise for improving diabetes outcomes. However, further longitudinal and experimental research is needed to confirm these relationships.

## Graphical abstract

Self‐efficacy emerged as the central psychological driver linking health literacy, social support and care quality to improved self‐care behaviours, glycaemic control (HbA1c) and health‐related quality of life in adults with Type 2 diabetes across 13 studies from seven countries.

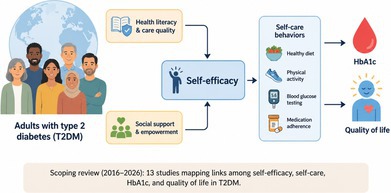

**Abbreviations**
Adj. diff.: adjusted mean difference
ANOVA: Analysis of variance
BMI: body mass index
CI: confidence interval
CINAHL: Cumulative Index to Nursing and Allied Health Literature
DMSE: Diabetes management self‐efficacy
DMSES: Diabetes Management Self‐Efficacy Scale
DSM: Diabetes Self‐Management
EQ‐5D: EuroQol 5‐Dimension
GSE‐6: General Self‐Efficacy Scale
HbA1c: haemoglobin A1c
HRQoL: health‐related quality of life
JBI: Joanna Briggs Institute
MeSH: Medical Subject Headings
NDS: nurse‐led diabetic service
NEPHSP: National Essential Public Health Services Program
ns: not statistically significant
NSSMP: nurse‐led smartphone‐based self‐management program
OR: Odds Ratio
PACIC: perceived care quality
PCC: patient‐centred care
PRISMA‐ScR: Preferred reporting items for systematic reviews and meta‐analysis extension for scoping review
QoL: quality of life
RCT: randomised controlled trial
SCT: social cognitive theory
SDM: shared decision‐making
SED: Self‐Efficacy for Diabetes
SE‐focused: self‐efficacy–focused
SMS4BG: text message‐based diabetes self‐management support program
Std. Est.: Standardised Estimate
T2DM: type 2 diabetes mellitus
VAS: VAS: visual analogue scale
Δ: change

## 1 Introduction

Type 2 diabetes mellitus (T2DM) is a widespread chronic metabolic disorder and a major contributor to global morbidity and mortality [1, 2]. Despite advances in pharmacological therapies and clinical guidelines, the global burden of T2DM continues to rise, with significant implications for healthcare costs, life expectancy, and psychosocial well‐being [3, 4, 5, 6, 7, 8, 9]. Glycemic control, most commonly assessed by haemoglobin A1c (HbA1c), is central to effective diabetes management. Improved glycaemic control is associated with reduced microvascular complications and, to a lesser extent, macrovascular complications [3]. However, many adults with T2DM do not meet recommended glycaemic targets, indicating that pharmacotherapy alone does not ensure optimal outcomes [10, 11]. This gap highlights the critical role of diabetes self‐care behaviours, such as dietary adherence, physical activity, blood glucose self‐monitoring, foot care, and medication adherence, in achieving and maintaining glycaemic control and preventing complications [12, 13].

Self‐care in T2DM is multifaceted, requiring individuals to engage in ongoing behavioural tasks shaped by cognitive, emotional, social and environmental influences [12]. Among these factors, self‐efficacy, defined as confidence in the ability to perform specific health behaviours has emerged as a key psychological construct in diabetes management. Higher diabetes‐related self‐efficacy is consistently linked to better adherence to self‐care behaviours, improved metabolic outcomes and enhanced quality of life [14, 15]. For instance, greater self‐efficacy is strongly associated with both improved diabetes self‐care and lower HbA1c levels in adults with T2DM, and is the psychosocial factor most closely related to self‐care and glycaemic control in Turkish patients [16]. Recent research also suggests that broader constructs, such as self‐care agency, are positively associated with quality of life among adults with diabetes [17].

Social and psychological determinants, such as psychological distress, health literacy and social support, influence diabetes outcomes both directly and indirectly. Walker et al. demonstrated that lower psychological distress, greater social support, and higher self‐efficacy are associated with improved self‐care [18]. Psychosocial determinants also have direct associations with glycaemic control, independent of self‐care behaviours. A meta‐analysis by Marciano et al. found that higher health literacy is linked to better diabetes knowledge, more appropriate self‐care and improved glycaemic control [11]. Ghaffari‐fam et al. reported that health literacy and self‐care behaviours together affect health‐related quality of life among Iranian adults with T2DM [8]. Evidence further suggests that integrated interventions combining lifestyle, educational and psychological components [19, 20] are more effective than single‐component interventions for improving mental health, self‐management, and diabetes outcomes [3, 21]. Collectively, these findings indicate that self‐efficacy and self‐care are embedded within a broader psychosocial, literacy and social‐determinant context that shapes glycaemic control [22].

Quality of life (QoL) has become a central outcome in diabetes care, shifting the focus from mortality and morbidity indicators to patients' subjective perceptions of physical, psychological, social, and environmental well‐being [23]. Multiple studies have demonstrated that suboptimal self‐care behaviours and poor glycaemic control are associated with reduced health‐related quality of life in individuals with T2DM [24, 25]. In an Iranian cohort, self‐care behaviours, particularly nutritional self‐care, blood glucose self‐management and self‐medication, were significant predictors of QoL, with nutritional self‐care showing the strongest association [26]. Similarly, nonadherence to key self‐care behaviours such as exercise, foot care and smoking cessation was associated with lower EQ‐5D indices and greater impairment across multiple QoL domains, including mobility, self‐care, usual activities, pain and anxiety or depression [27]. Additional studies indicate that social support, health literacy and self‐management behaviours are linked to both glycaemic control and QoL, particularly among older adults [14, 28]. Educational and diabetes self‐management interventions have also been shown to enhance self‐care behaviours, improve health beliefs and increase QoL, along with modest improvements in anthropometric and metabolic parameters [3, 21].

Despite the expanding body of evidence, existing studies are heterogeneous in terms of settings, populations, conceptual frameworks and outcome measures, and often examine only a subset of the relationships among self‐efficacy, self‐care, glycaemic control and QoL. Many investigations focus on one or two of these variables, use varying operational definitions, or are limited to specific cultural or healthcare contexts, complicating the integration of findings and the development of comprehensive conclusions [12, 13, 15]. To date, no scoping review has systematically mapped and synthesised the extent, range, and nature of research examining how self‐efficacy and self‐care behaviours simultaneously relate to glycaemic control and quality of life in adults with T2DM across diverse health systems and sociocultural settings.

Given the conceptual complexity of the interrelationships among self‐efficacy, self‐care, glycaemic control and HRQoL, and the diversity of study designs, populations and measurement approaches in the existing literature, a scoping review was deemed most appropriate. Unlike systematic reviews, scoping reviews are designed to map the extent, range and nature of available evidence without requiring homogeneity of study designs or outcomes, making this approach uniquely suited to characterising the emerging evidence base and identifying knowledge gaps [29, 30, 31]. Specifically, no prior scoping review has systematically mapped the simultaneous relationships among all four constructs across diverse settings. Therefore, the objective of this scoping review is to map the available literature on the relationships among self‐efficacy, self‐care, glycaemic control and quality of life in adults with T2DM.

## 2 Materials and Methods

### 2.1 Study Design

This study employed a scoping review design. The methodological framework established by Arksey and O'Malley [29], and subsequently refined by Levac et al., which guided the sequential stages of the review process, including scope formulation, database identification, study selection, data charting and evidence synthesis [30]. The Joanna Briggs Institute (JBI) guidance informed the methodological conduct of the review, including the management of inclusion criteria and the approach to narrative synthesis [31]. Reporting followed the Preferred Reporting Items for Systematic Reviews and Meta‐Analyses Extension for Scoping Reviews (PRISMA‐ScR). The scoping review approach was chosen for its systematic approach to map existing evidence and identify knowledge gaps regarding the influence of self‐efficacy and self‐care behaviours on glycaemic control in adults with T2DM. No protocol for this scoping review was prospectively registered before commencement. While prospective registration via platforms such as OSF or PROSPERO is increasingly recommended as a best practice to enhance transparency, it is not currently mandated for scoping reviews, unlike for systematic reviews and meta‐analyses. In line with JBI and PRISMA‐ScR (preferred reporting items for systematic reviews and meta‐analysis extension for scoping review) guidance, we did not perform a formal risk‐of‐bias assessment, as the primary aim was to map the existing evidence rather than to conduct a quantitative synthesis.

### 2.2 Eligibility Criteria

#### 2.2.1 Inclusion Criteria

- Studies published in English from January 2016 to March 2026
- Studies including adults aged 18 years or older diagnosed with T2DM
- Studies addressing at least one of the following constructs: diabetes management self‐efficacy, self‐care behaviours, glycaemic control (HbA1c) or health‐related quality of life
- All study designs were eligible, including randomised controlled trials (RCTs), cross‐sectional studies, longitudinal studies and quasi‐experimental studies

#### 2.2.2 Exclusion Criteria

- Studies exclusively involving participants with Type 1 diabetes or gestational diabetes.
- Studies that did not include at least one of the following as a primary variable: self‐efficacy, self‐care behaviours, glycaemic control (HbA1c) or health‐related quality of life (HRQoL).
- Conference abstracts, case reports, editorials, expert opinions and studies duplicating data from the same dataset without novel contributions.

### 2.3 Information Sources

An electronic database search was conducted across the following databases: PubMed/MEDLINE, Scopus, ScienceDirect and CINAHL (Cumulative Index to Nursing and Allied Health Literature). The search covered the period from January 2016 to March 2026 and was limited to English‐language publications.

### 2.4 Search Strategy

The search strategy was developed in accordance with specialist information retrieval principles. A pilot search was conducted before finalising the full approach. Boolean operators (AND, OR) were used to combine Medical Subject Headings (MeSH) terms and free‐text keywords. For example, the PubMed search string included: (“type 2 diabetes mellitus”[MeSH] OR “T2DM”) AND (“self‐efficacy”[MeSH] OR “diabetes self‐efficacy”) AND (“self‐care”[MeSH] OR “self‐management”) AND (“glycemic control” OR “HbA1c” OR “haemoglobin A1c”) AND (“quality of life”[MeSH] OR “HRQoL”). The complete database‐specific search strings are provided in Table S1.

#### 2.4.1 Study Selection Process and Data Extraction

**FIGURE 1:**
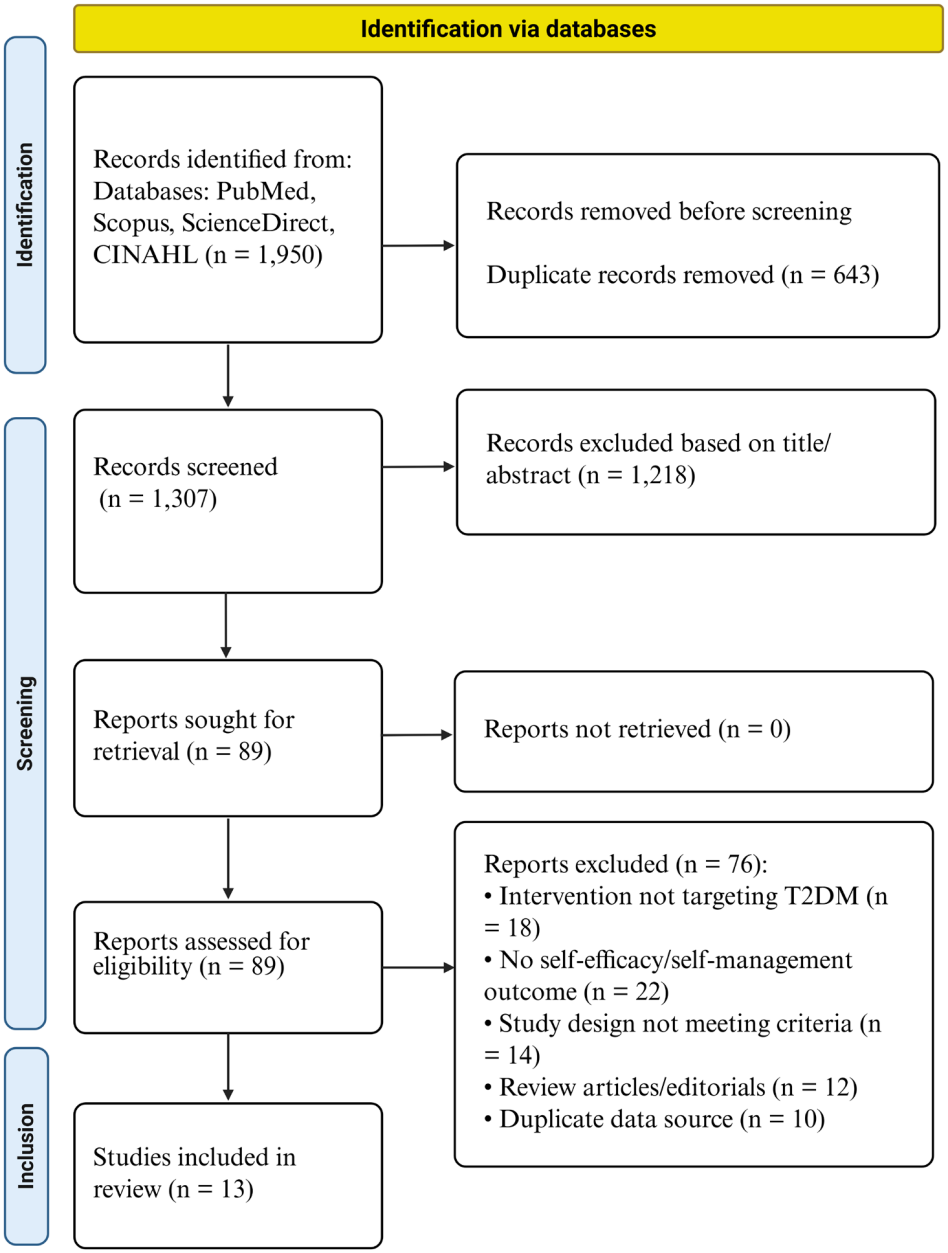
PRISMA‐ScR flow diagram of the study selection process. PRISMA‐ScR: Preferred reporting items for systematic reviews and meta‐analysis extension for scoping review.

The study selection process comprised two stages: title screening and abstract screening. Two authors (O.A. and F.H.) independently screened titles/abstracts and full texts against the eligibility criteria. Duplicates were removed prior to screening, and disagreements were resolved by discussion with a third author (S.D.). This approach ensured a rigorous evaluation, and the selection process is illustrated in the PRISMA‐ScR flow diagram (Figure 1). Subsequently, data extraction was conducted using a standardised, pre‐designed data charting form developed collaboratively by the review team and independently applied by two reviewers. The extracted variables included author and year, study purpose, country, sample size, study design and main findings. A completed PRISMA‐ScR reporting checklist is provided in Table S2.

HRQoL outcomes were operationalised across studies using validated, standardised instruments. Among the most frequently reported, the EuroQol Five‐Dimension (EQ‐5D) tool measures health status across five dimensions: mobility, self‐care capacity, usual activities, pain/discomfort and anxiety/depression.

### 2.5 Critical Appraisal and Synthesis of Evidence

Consistent with PRISMA‐ScR guidelines for scoping reviews [45], this review did not conduct a formal assessment of methodological quality or risk of bias for the included studies, a process that is typically reserved for systematic reviews. In the absence of a formal risk‐of‐bias assessment, which is consistent with established scoping review methodology, study design type (e.g., RCT, cross‐sectional) and publication in peer‐reviewed journals were considered descriptively when interpreting the strength and direction of findings. No formal credibility scoring or quality ranking was applied to individual studies. The primary aim was to map the scope of available evidence rather than to appraise the quality of individual studies for meta‐analysis. Therefore, both reviews and primary studies were included without quantitative analysis, and any duplication is not a concern. Data were synthesised descriptively to categorise interventions and to summarise their effects on patient outcomes. Data were synthesised descriptively using a narrative approach to categorise study characteristics, intervention types, and their effects on self‐efficacy, self‐care behaviours, glycaemic control and quality‐of‐life outcomes in adults with type 2 diabetes. The heterogeneity of included study designs, ranging from cross‐sectional studies to RCTs, was addressed through a descriptive narrative synthesis rather than quantitative pooling. Findings from each design were interpreted in light of the inferential limitations inherent to that design: cross‐sectional studies were interpreted as evidence of association only, longitudinal and quasi‐experimental studies as suggestive of temporal relationships, and RCTs as the most robust evidence of intervention effectiveness. This design‐sensitive approach to synthesis is consistent with JBI guidance for scoping reviews.

## 3 Results

### 3.1 Overview of Included Studies

A systematic search of four electronic databases, PubMed/MEDLINE, Scopus, ScienceDirect and CINAHL, identified 13 studies that met the predefined eligibility criteria. These studies, published between 2016 and 2026, enrolled 5784 adults with T2DM. The study encompassed countries such as the United States, China, Taiwan, Thailand, Singapore, Iran and New Zealand. This geographical diversity underscores the global scope of the review and facilitates cross‐cultural integration of the evidence.

The included studies used various designs: five randomised controlled trials (RCTs), six cross‐sectional studies, one quasi‐experimental pretest/posttest study and one longitudinal study with a one‐year follow‐up. Specifically, the quasi‐experimental design involved pre‐ and post‐intervention assessments without randomisation, whereas the cross‐sectional studies collected data at a single point in time. Sample sizes ranged from 106 to 1577 participants. Table 1 provides a detailed summary of all included study characteristics. Several important themes evolved from the data.

**TABLE 1:** Characteristics of included studies (*n* = 13).

Author (Year)	Purpose	Settings	Sample size	Study design	Main findings
**Cross sectional study (*n* = 6)**					
Williams et al. (2016) [32]	To evaluate the relationship between patient‐centred care (PCC), diabetes self‐care, glycaemic control and quality of life in adults with T2DM	USA	*n* = 615	Cross‐sectional study	PCC was significantly associated with mental QoL and most self‐care behaviours (medication adherence, diet, blood sugar testing, foot care) but was not significantly associated with glycaemic control after covariate adjustment
Li et al. (2024) [33]	To examine the associations between perceived care quality (PACIC), self‐care behaviours and glycaemic control in Chinese adults with T2DM under the National Essential Public Health Services Program (NEPHSP)	China	*n* = 1577	Cross‐sectional study	Higher perceived care quality was significantly associated with improved self‐care behaviours and better glycaemic control; self‐care behaviours mediated over half of this effect on HbA1c.
Nilmart et al. (2025) [34]	To identify distinct patient subgroups based on glycemic control, self‐efficacy and self‐management in patients with T2DM using cluster analysis	Thailand	*n* = 440	Cross‐sectional study	Four distinct patient clusters were identified: moderate profile, underperforming, high performers, and high‐risk; findings demonstrated marked heterogeneity across clinical, behavioural and psychological profiles, underscoring the need for tailored interventions
Zhao et al. (2024) [35]	To test the serial mediation roles of empowerment and self‐care activities in the associations between health literacy and QoL and between health literacy and HbA1c in people with T2DM	China	*n* = 319	Cross‐sectional study	Serial mediation of empowerment and self‐care activities in the health literacy–quality of life relationship (Std. Est. = 0.046) and health literacy–HbA1c relationship (Std. Est. = 0.045) were both statistically significant
Ting et al. (2025) [36]	To explore the dynamics of self‐efficacy, psychological resilience and self‐management on QoL in T2DM patients from a positive psychology perspective	China	*n* = 408	Cross‐sectional study	Self‐efficacy had a significant direct and indirect effect on QoL, with resilience serving as a partial mediator (accounting for 43.1% of the effect); self‐management level significantly moderated the relationships between self‐efficacy, resilience and QoL
Hurst et al. (2020) [37]	To investigate the impact of diabetes self‐management, management self‐efficacy and diabetes knowledge on glycaemic control in Thai patients with T2DM	Thailand	*n* = 700	Multi‐center cross‐sectional study	Diabetes management self‐efficacy (DMSE) was the strongest independent predictor of glycaemic control after full adjustment (OR = 2.67), surpassing both self‐management behaviours and diabetes knowledge
**Longitudinal study (*n* = 1)**					
Hsu et al. (2018) [38]	To test a hypothesised model addressing the influencing pathways among personal characteristics, social support, diabetes distress and self‐care behaviours to HbA1c and quality of life	Taiwan	*n* = 382	Longitudinal study (1‐year follow‐up)	Quality of life directly influenced HbA1c; self‐care behaviours directly affected QoL and indirectly affected HbA1c through quality of life; baseline diabetes distress negatively predicted quality of life at 12 months
**Quasi‐experimental study (*n* = 1)**					
Kaveh et al. (2022) [39]	To examine the effects of a Social Cognitive Theory (SCT)‐based training program with follow‐up home visits on self‐management, glycaemic index and QoL in Iranian patients with T2DM	Iran	*n* = 106	Quasi‐experimental (pre‐test/post‐test design)	The SCT‐based intervention significantly improved all SCT constructs, self‐management behaviours, QoL and glycaemic control; HbA1c decreased from 8.29 to 6.28 in the intervention group (*p* < 0.001)
**Randomised Controlled Trials (*n* = 5)**					
Jiang et al. (2022) [40]	To evaluate the effectiveness of a nurse‐led smartphone‐based self‐management program (NSSMP) in improving outcomes for poorly controlled T2DM	Singapore	*n* = 114	Two‐arm RCT	NSSMP was as effective as the existing nurse‐led diabetic service (NDS) in improving self‐efficacy, diabetes self‐care, health‐related QOL and reducing HbA1c; significant group difference was found only in blood glucose testing activity
Dobson et al. (2018) [41]	To determine the effectiveness of a theoretically based, individually tailored text message‐based diabetes self‐management support program (SMS4BG) in adults with poorly controlled diabetes	New Zealand	*n* = 366	Two‐arm parallel RCT (9‐month)	SMS4BG resulted in significantly greater reductions in HbA1c compared to usual care (adjusted mean difference = −4.23 mmol/mol, *p* = 0.007); significant improvements were also noted in foot care, overall diabetes support, and health status
Lyu et al. (2021) [42]	To evaluate the effects of a nurse‐led web‐based transitional care program on glycaemic control and quality of life post‐hospital discharge in patients with T2DM	China	*n* = 116	RCT	The web‐based transitional care program significantly improved glycaemic control (ΔHBA1c = 2.87, *p* < 0.01) and quality of life (Δ7.69, *p* < 0.01); self‐efficacy and treatment adherence were identified as significant mediators of these effects
Jiang et al. (2019) [43]	To evaluate the effectiveness of a self‐efficacy‐focused structured education program on metabolic and psychosocial outcomes in T2DM adults without insulin therapy	China	*n* = 265	Multicentre parallel RCT	The program significantly improved HbA1c, BMI, waist circumference, diastolic blood pressure, self‐efficacy, self‐management behaviours, and diabetes knowledge at 6‐month follow‐up compared to the control group (all *p* < 0.001)
Zhang et al. (2024) [44]	To evaluate the effectiveness of a shared decision‐making (SDM) informed dietary intervention based on digital health technology in older adults with T2DM	China	*n* = 124	Two‐arm parallel RCT	The SDM‐based digital dietary intervention significantly improved HbA1c, fasting plasma glucose, diastolic blood pressure, self‐management behaviour, and self‐efficacy compared with the control group at 3‐month follow‐up (*p* < 0.05)

Abbreviations: BMI, body mass index; DMSE, Diabetes management self‐efficacy; NDS, nurse‐led diabetic service; NEPHSP, National Essential Public Health Services Program; NSSMP, nurse‐led smartphone‐based self‐management program; PACIC, perceived care quality; PCC, patient‐centred care; QoL, quality of life; RCT, randomised controlled trial; SCT, social cognitive theory; SDM, shared decision‐making; SMS4BG, text message‐based diabetes self‐management support program; T2DM, type 2 diabetes mellitus.

### 3.2 Theme 1: The Role of Self‐Care Behaviours as a Mediating Pathway Between Care Quality and Glycaemic Control

Multiple studies have demonstrated that self‐care behaviours are a central mediator linking care quality, patient‐centred support and glycaemic outcomes. Table 2 presents the principal quantitative findings from these studies.

**TABLE 2:** Key quantitative findings on self‐care behaviours, glycaemic control and quality of life.

Author (Year)	Direction of effect	Outcomes measured	Main statistical findings
Williams et al. (2016) [32]	PCC → self‐care	HbA1c, QoL (physical and mental component score), Self‐care	PCC → medication adherence: *β* = 0.12 (95% CI: 0.08–0.17); PCC → mental QoL: *β* = 0.09 (95% CI: 0.04–0.14); PCC → HbA1c: *β* = −0.03, ns
Li et al. (2024) [33]	PACIC → self‐care → HbA1c	HbA1c, Self‐care composite score	PACIC → self‐care: *β* = 0.294 (95% CI: 0.233–0.354); PACIC → HbA1c: *β* = −0.109 (95% CI: −0.192 to −0.026); Self‐care mediated 50.41% of effect (*p* < 0.05)
Nilmart et al. (2025) [34]	Heterogeneity across patient profiles	HbA1c, Self‐efficacy, Self‐management	4 clusters; High‐risk cluster: HbA1c = 9.4%, self‐efficacy = 56/100, self‐management = 34/91
Zhao et al. (2024) [35]	Serial mediation	QoL, HbA1c (serial mediation)	Health literacy → QoL via empowerment + self‐care: Std. Est. = 0.046 (*p* = 0.019); Health literacy → HbA1c via same path: Std. Est. = 0.045 (*p* = 0.005)
Ting et al. (2025) [36]	Self‐efficacy → resilience → QoL; moderated by self‐management	QoL	Self‐efficacy → QoL direct: path c’ = −0.753 (*p* < 0.05); Resilience mediates 43.1%; Self‐management moderates (*p* = 0.002)
Hurst et al. (2020) [37]	Self‐efficacy dominant over self‐management	HbA1c control	DMSE → HbA1c: OR = 2.67 (95% CI: 2.20–3.25, *p* < 0.001); DSM → HbA1c after DMSE adjustment: OR = 1.11 (ns)
Hsu et al. (2018) [38]	Self‐care → QoL → HbA1c	HbA1c, QoL, Self‐care	6‐month self‐care → 12‐month QoL (direct, *p* < 0.01); 12‐month QoL → 12‐month HbA1c (direct, *p* < 0.01); Self‐care → HbA1c indirect: −0.043 (*p* = 0.046)
Kaveh et al. (2022) [39]	SCT intervention → all outcomes	HbA1c, QoL, SCT constructs	HbA1c: 8.29% → 6.28% in intervention group (*p* < 0.001); QoL change: +16.90 ± 7.39 vs. +0.57 ± 2.99 (*p* < 0.001)
Jiang et al. (2022) [40]	NSSMP equivalent to NDS	HbA1c, Self‐efficacy, HRQoL	No significant between‐group differences except blood glucose testing activity (F = 4.742, *p* = 0.015)
Dobson et al. (2018) [41]	SMS4BG → HbA1c and foot care	HbA1c, Foot care, EQ‐5D VAS	HbA1c adjusted mean difference: −4.23 mmol/mol (95% CI: −7.30 to −1.15, *p* = 0.007); Foot care: +0.85 (*p* < 0.001)
Lyu et al. (2021) [42]	Web‐based care → HbA1c + QoL via self‐efficacy	HbA1c, QoL	ΔHBA1c: −2.87 (*p* < 0.01); ΔQoL: +7.69 (*p* < 0.01); Self‐efficacy mediated: indirect = 0.18 (*p* < 0.05)
Jiang et al. (2019) [43]	SE‐focused education → HbA1c	HbA1c, Self‐efficacy, Self‐management	HbA1c adjusted mean difference: −0.740 (95% CI: −1.045 to −0.434, *p* < 0.001); Self‐efficacy: +0.517 (*p* < 0.001)
Zhang et al. (2024) [44]	SDM digital intervention → HbA1c + self‐efficacy	HbA1c, Fasting plasma glucose, Self‐efficacy	HbA1c: 6.92 ± 1.03 vs. 7.58 ± 1.13 (*t* = 3.298, *p* < 0.001); Self‐efficacy time × group: *F* = 7.127, *p* < 0.001

Abbreviations: →, directional pathway; CI, confidence interval; DMSE, Diabetes management self‐efficacy; DSM, Diabetes Self‐Management; EQ‐5D, EuroQol 5‐Dimension; HbA1c, haemoglobin A1c; HRQoL, health‐related quality of life; ns, nonsignificant; NDS, Nurse‐led Diabetic Service; NSSMP, nurse‐led smartphone‐based self‐management program; OR, Odds Ratio; PACIC, perceived care quality; PCC, patient‐centered care; QoL, quality of life; SCT, social cognitive theory; SDM, shared decision‐making; SE‐focused, self‐efficacy‐focused; SMS4BG, text message‐based diabetes self‐management support program; Std. Est., Standardised Estimate; T2DM, type 2 diabetes mellitus; VAS, visual analog scale; Δ, change.

Williams et al., in a cross‐sectional study of 615 US adults, found that patient‐centred care (PCC) was significantly associated with medication adherence (*β* = 0.12, 95% CI: 0.08–0.17), general diet (*β* = 0.12, 95% CI: 0.07–0.17), blood glucose testing (*β* = 0.09, 95% CI: 0.04–0.15), foot care (*β* = 0.12, 95% CI: 0.07–0.18) and the mental component of quality of life (QoL) (*β* = 0.09, 95% CI: 0.04–0.14) [32]. In contrast, PCC was not significantly associated with glycaemic control after full covariate adjustment (*β* = −0.03 [negative direction], 95% CI: −0.07 to 0.01, *P* = ns). Notably, PCC was also significantly but inversely associated with the physical component of QoL (*β* = −0.03, 95% CI: −0.05 to −0.01, *p* < 0.05), suggesting that greater patient‐centredness was paradoxically associated with lower physical QoL scores in this sample, possibly reflecting a higher burden of self‐management awareness among more engaged patients.

Li et al. reported that the overall self‐care behaviour score was significantly and negatively associated with HbA1c in the fully adjusted model (*β* = −0.197 [negative direction, indicating that higher self‐care scores were associated with lower HbA1c], 95% CI: −0.263 to −0.132, *p* < 0.001) [33]. Causal mediation analysis further demonstrated that self‐care behaviours statistically accounted for 50.41% of the association between perceived care quality (PACIC) and glycaemic control in this cross‐sectional analysis (*p* < 0.05), with individual self‐care behaviours showing the strongest associations for no current smoking (*β* = −0.590, 95% CI: −0.827 to −0.353, *p* < 0.001), regular health check‐ups (*β* = −0.405, 95% CI: −0.591 to −0.219, *p* < 0.001) and regular blood glucose self‐monitoring (*β* = −0.369, 95% CI: −0.620 to −0.118, *p* = 0.004).

Hsu et al. contributed the only longitudinal pathway evidence in this review, using structural equation modelling to show that self‐care behaviours at 6 months exerted a significant positive direct effect on 12‐month QoL, and a significant negative indirect effect on 12‐month HbA1c through QoL (indirect path coefficient = −0.043, *p* = 0.046) [38], indicating that better self‐care was associated with improved QoL, which in turn was associated with lower HbA1c. Baseline diabetes distress was a significant negative predictor of 12‐month QoL (direct path, *p* < 0.01), while baseline social support exerted significant indirect positive effects on QoL mediated through both diabetes distress and self‐care behaviours (*p* < 0.001).

### 3.3 Theme 2: Diabetes Management Self‐Efficacy as a Key Factor Associated With Glycaemic Control

Hurst et al. demonstrated that Diabetes Management Self‐Efficacy (DMSE) was the strongest independent factor positively associated with HbA1c control in this multivariable cross‐sectional analysis (OR = 2.67, 95% CI: 2.20–3.25, *p* < 0.001) [35]. This indicates that patients with higher self‐efficacy scores had 2.67 times greater odds of achieving good glycaemic control (HbA1c ≤ 7%) compared to those with lower scores. By comparison, the unadjusted bivariate OR for DMSE was 2.20 (95% CI: 1.97–2.46, *p* < 0.001), and for diabetes self‐management (DSM) was 1.62 (95% CI: 1.46–1.80, *p* < 0.001). After mutual adjustment, DSM was attenuated to OR = 1.11 (*p* = 0.176, nonsignificant), while diabetes knowledge was nonsignificant across all models.

In Iran, Kaveh et al. found that a Social Cognitive Theory (SCT)‐based training program reduced HbA1c from 8.29% to 6.28% (a 2.01 percentage‐point reduction, *p* < 0.001) in the intervention group, compared to no significant change in controls, alongside significant improvements in self‐efficacy (change: +7.16 ± 0.63 vs. +0.02 ± 0.51, *p* < 0.001) and QoL (+16.90 ± 7.39 vs. +0.57 ± 2.99, *p* < 0.001) [39]. Jiang et al. (2019) reported similar findings via a multicentre RCT of 265 adults with T2DM, with the self‐efficacy‐focused structured education program producing significant improvements in HbA1c (adjusted mean difference: −0.740, 95% CI: −1.045 to −0.434, *p* < 0.001), self‐management behaviours (+13.801, *p* < 0.001) and diabetes knowledge (+3.204, *p* < 0.001) at 6 months, with equal effectiveness across educational levels [43]. It is important to note that the included studies used heterogeneous self‐efficacy measures, including the Diabetes Management Self‐Efficacy scale (DMSE), the Self‐Efficacy for Diabetes scale (SED), the Diabetes Management Self‐Efficacy Scale (DMSES) and the General Self‐Efficacy scale (GSE‐6) (Table S3). These instruments differ in their conceptual scope: DMSE, SED, and DMSES are diabetes‐specific measures, whereas GSE‐6 is a general self‐efficacy tool not specific to diabetes management. This measurement heterogeneity limits direct cross‐study comparisons of effect sizes and may partly explain the variability in findings across studies. Future research should adopt standardised, diabetes‐specific self‐efficacy measures to enable more meaningful comparisons. Detailed information on the self‐efficacy instruments used in each study, including scale type, number of items, scoring range and psychometric properties, is provided in Table S3.

### 3.4 Theme 3: Effectiveness of Digital Health Interventions

Dobson et al. conducted the largest digital intervention RCT in this review (*n* = 366) [39]. They found that the SMS4BG (text message‐based diabetes self‐management support) program, an individually tailored nine‐month intervention, led to a significantly greater reduction in HbA1c than usual care (adjusted mean difference: −4.23 mmol/mol; 95% CI: −7.30 to −1.15; *p* = 0.007). The program also significantly improved foot care (adjusted difference: 0.85, *p* < 0.001) and health status as measured by the EQ‐5D VAS (+4.38, *p* = 0.03). By comparison, Jiang et al. (2022) reported no significant between‐group differences in HbA1c at any time point (6‐month mean difference: −0.118, 95% CI: −0.767 to 0.531, *p* = 0.719), self‐efficacy (GSE interaction effect: *F* = 2.982, *p* = 0.053) or HRQoL (Audit of Diabetes‐Dependent QoL, generic present QoL interaction: *F* = 0.066, *p* = 0.936) when comparing the NSSMP smartphone application with the existing nurse‐led diabetic service. The only significant between‐group difference was in blood glucose testing activity (*F* = 4.742, *p* = 0.015), favouring the control group, likely reflecting the more intensive glucose monitoring follow‐up inherent to the NDS protocol. [40]. This lack of difference was likely due to a high‐quality, intensive comparator and reduced statistical power (actual *n* = 114 versus target *n* = 128) after COVID‐19 disruptions. Lyu et al. found that a nurse‐led web‐based transitional care program significantly reduced HbA1c (ΔHbA1c = −2.87, *p* < 0.01) and improved quality of life (ΔQoL = +7.69, *p* < 0.01) [42]. Self‐efficacy and treatment adherence were significant mediators for both outcomes (*R* ^2^ for HbA1c = 52.5%; *R* ^2^ for QoL = 34.2%). An expanded summary of digital interventions and comparator self‐efficacy‐based education programs is presented in Table S4.

### 3.5 Theme 4: Model‐Based Evidence on Empowerment, Resilience and Psychological Pathways

Three cross‐sectional studies employed structural equation modelling and moderated mediation analysis to examine hypothesised psychological and social pathways connecting self‐efficacy, self‐care and health outcomes in adults with T2DM. While these analyses provide valuable model‐based insights, the cross‐sectional nature of the data precludes confirmation of causal directionality.

Three studies examined how psychological and social factors relate to confidence in managing diabetes (self‐efficacy), personal healthcare (self‐care) and health outcomes among people with type 2 diabetes, revealing complex relationships.

Zhao et al. conducted a cross‐sectional study (*n* = 319) to test a serial mediation model using structural equation modelling [35]. Results indicated that communicative health literacy was the primary contributor to the overall health literacy construct. Both the direct effect of health literacy on QoL (Std. Est. = 0.147, *p* = 0.018) and the serial mediation of empowerment and self‐care activities in the health literacy–QoL pathway (Std. Est. = 0.046, 95% CI: 0.085–0.008, *p* = 0.019) were statistically significant. Similarly, the serial mediation of empowerment and self‐care activities in the health literacy–HbA1c pathway was significant (Std. Est. = 0.045, 95% CI: 0.077–0.013, *p* = 0.005), collectively accounting for 99.32% of the total indirect effect from health literacy to QoL.

Ting et al., in a cross‐sectional study of 408 patients with T2DM in China, found that self‐efficacy had a significant direct effect on QoL (path c' = −0.753, 95% CI: −1.476 to −0.031, *p* < 0.05) and an indirect effect mediated by resilience (indirect effect = −0.781, 95% CI: −1.283 to −0.410, *p* < 0.001), with resilience accounting for 43.1% of the total effect [36]. Furthermore, moderated mediation analysis revealed that self‐management significantly moderated both the self‐efficacy–QoL relationship (interaction coefficient = −0.075, 95% CI: −0.122 to −0.028, *p* = 0.002) and the resilience–QoL pathway (−0.007, 95% CI: −0.013 to −0.002, *p* = 0.012), indicating that higher self‐management amplified the beneficial effects of both self‐efficacy and resilience on QoL. The final moderated mediation model explained 31.2% of the variance in QoL (*R* ^2^ = 0.3123).

### 3.6 Theme 5: Patient Subgroup Heterogeneity and Predictors of Self‐Efficacy and Self‐Management

Two studies specifically examined heterogeneity among T2DM patients in terms of behavioural and clinical profiles, providing important insights for tailored interventions. Nilmart et al., using k‐means cluster analysis in a cross‐sectional study of 440 Thai patients with T2DM, identified four distinct subgroups based on HbA1c, self‐efficacy, and self‐management scores [34]: (1) Cluster 1 “Moderate Profile” (*n* = 124; HbA1c = 7.9%, self‐efficacy = 70/100, self‐management = 47/91); (2) Cluster 2 “Underperforming” (*n* = 136; HbA1c = 8.7%, self‐efficacy = 79, self‐management = 40); (3) Cluster 3 “High Performers” (*n* = 135; HbA1c = 7.5%, self‐efficacy = 84, self‐management = 51); and (4) Cluster 4 “High Risk” (*n* = 45; HbA1c = 9.4%, self‐efficacy = 56, self‐management = 34). Analysis of variance (ANOVA) confirmed significant between‐cluster differences across all three clustering variables (*p* < 0.001). Notably, Cluster 2 patients paradoxically exhibited high self‐efficacy alongside poor self‐management and glycaemic control, suggesting that perceived confidence does not invariably translate into effective self‐care behaviour in the absence of adequate practical support and skills. Regression analysis confirmed that the overall model explained 18.2% of the variance in HbA1c (adjusted *R* ^2^ = 0.182), with treatment regimen, dyslipidaemia and education level as significant independent predictors.

Hurst et al. found that over half of Thai patients did not achieve good blood sugar control [37]. Confidence in managing diabetes (self‐efficacy) was strongly associated with better blood sugar levels on its own, even after controlling for self‐care behaviours, suggesting that confidence matters independently for health outcomes.

## 4 Discussion

### 4.1 Self‐Care as the Central Mediating Mechanism

A fundamental and cross‐cutting conclusion emerging from this review is that self‐care behaviours function as the primary behavioural intermediary through which care quality, patient‐centred support and psychological resources translate into improved glycaemic control. Li et al. provided the most robust quantitative evidence for this mediation, establishing that self‐care behaviours accounted for over half (50.41%) of the total effect of perceived care quality on HbA1c through a causal mediation analysis, independent of demographic and clinical confounders [33]. This is consistent with the Chronic Care Model, which posits that productive interactions between an informed, activated patient and a prepared, proactive care team represent the fundamental mechanism of chronic disease outcomes. Williams et al. reinforced this framework by demonstrating that PCC significantly facilitated most self‐care domains but did not show a direct association with glycaemic control, indicating that improvements in care quality may require adequate behavioural translation before metabolic benefits become observable, though causal directionality cannot be confirmed from cross‐sectional evidence alone [32].

The longitudinal evidence provided by Hsu et al. further reveals an important temporal dimension of this pathway, demonstrating that self‐care behaviours at 6 months exerted a significant indirect effect on HbA1c at 12 months mediated through QoL [38]. This pathway of self‐care → QoL → HbA1c challenges the assumption of a direct, linear relationship between self‐care behaviour and glycaemic reduction, instead positioning quality of life as both a therapeutic target and an intermediate mechanism. Patients with higher QoL may exhibit greater motivation, reduced diabetes distress, and greater willingness to sustain complex daily self‐care regimens, ultimately resulting in improved glycaemic control. These findings suggest that QoL warrants consideration as a primary endpoint in future research.

### 4.2 Primacy of Diabetes Management Self‐Efficacy Over Knowledge and Behaviour

Among all psychological and behavioural constructs examined, diabetes management self‐efficacy consistently emerged as the strongest independent predictor of glycaemic outcomes, frequently surpassing diabetes knowledge and self‐management behaviours when all three were entered simultaneously into multivariable models. The critical finding by Hurst et al., wherein the OR of self‐management behaviour for glycaemic control was substantially attenuated from OR = 1.69 to OR = 1.11 (nonsignificant) after adjustment for self‐efficacy, implies that a considerable portion of the behavioural pathway to glycaemic control operates through patients' confidence in their ability to execute self‐care tasks, not through the behaviours themselves in isolation [37]. This finding has direct implications for designing diabetes education programs: curricula that focus predominantly on knowledge acquisition and behavioural skill training, without simultaneously addressing patients' self‐efficacy beliefs, may be associated with limited improvements in glycaemic outcomes based on available evidence.

Two experimental studies support this point. Kaveh et al. found that a social cognitive theory‐based program cut HbA1c by 2.01 percentage points and raised self‐efficacy [39]. This program focused on Bandura's four sources of efficacy: mastery, vicarious learning, persuasion, and physiological states [46]. The work employed structured goal setting, peer modelling and nurse counselling. Jiang et al. (2019) showed that focusing on self‐efficacy helped patients regardless of educational background [43]. This makes such programs valuable for those with limited health literacy. Together, these studies support SCT‐based and self‐efficacy‐centred approaches as the heart of diabetes self‐management education and support.

### 4.3 Digital Health Interventions: Effectiveness Contingent on Design and Context

The synthesis of five digital intervention studies shows that effectiveness is not inherent to the digital modality. Instead, it depends on theoretical grounding, the degree of individualisation, intensity compared with the comparator and the targeted mechanisms. Dobson et al. reported the largest and most robust glycaemic benefit (HbA1c‐adjusted mean difference: −4.23 mmol/mol, *p* = 0.007) with an individually tailored text‐message program designed around behaviour change theory and patient preferences [41]. This intervention required only a standard mobile phone, no face‐to‐face contact and could be scaled for resource‐limited or dispersed populations. In contrast, Jiang et al. (2022) found no significant advantage of a smartphone app over a high‐quality nurse‐led service, highlighting that the benefit of digital tools is greatest when they substitute for or extend otherwise inaccessible care, not when they are redundant [40]. Lyu et al. and Zhang et al. showed that multicomponent digital platforms with peer support, personalised feedback, shared decision‐making and remote counselling can improve HbA1c, self‐efficacy, and QoL by addressing behavioural, cognitive and motivational aspects of self‐management [42, 44]. Not all digital interventions demonstrated superiority over standard care. Jiang et al. found no significant between‐group differences in HbA1c, self‐efficacy, or HRQoL when comparing a nurse‐led smartphone application with an established nurse‐led diabetes service [40]. This null finding is clinically informative: It suggests that the benefit of digital tools is contingent on the quality of the comparator, and that technology itself does not confer advantage when introduced alongside already high‐quality care. These inconsistencies across digital intervention studies underscore the importance of contextual factors, including healthcare infrastructure, intensity of comparator care and patient digital literacy in determining intervention effectiveness.

### 4.4 The Psychological Cascade: Health Literacy → Empowerment → Self‐Care → Outcomes

The serial mediation model of Zhao et al. makes a theoretically significant contribution by revealing a hierarchical psychological cascade through which health literacy influences clinical outcomes [44]. The finding that communicative health literacy, defined as the capacity to extract, process, and apply health information, was the dominant contributor to health literacy, and that empowerment and self‐care activities serially mediated its effects on both QoL and HbA1c, suggests that literacy interventions should emphasise interactive and dialogic methods rather than passive information delivery. Empowerment thus functions not just as an aspiration, but as a measurable mediator directly relevant to both subjective well‐being and objective metabolic control.

Ting et al. built on this model by showing that psychological resilience partly mediates the relationship between self‐efficacy and QoL, accounting for 43.1% of the effect [36]. They also found that self‐management acts as a key moderator. Higher self‐management strengthens both the self‐efficacy–QoL and resilience–QoL links. This points to a dynamic between behavioural skill and psychological strength. The lesson is clear: Working on just one psychological factor is not enough. Programmes that improve self‐efficacy, resilience, self‐management skills and empowerment together are more likely to produce lasting results in psychological and clinical outcomes.

**FIGURE 2:**
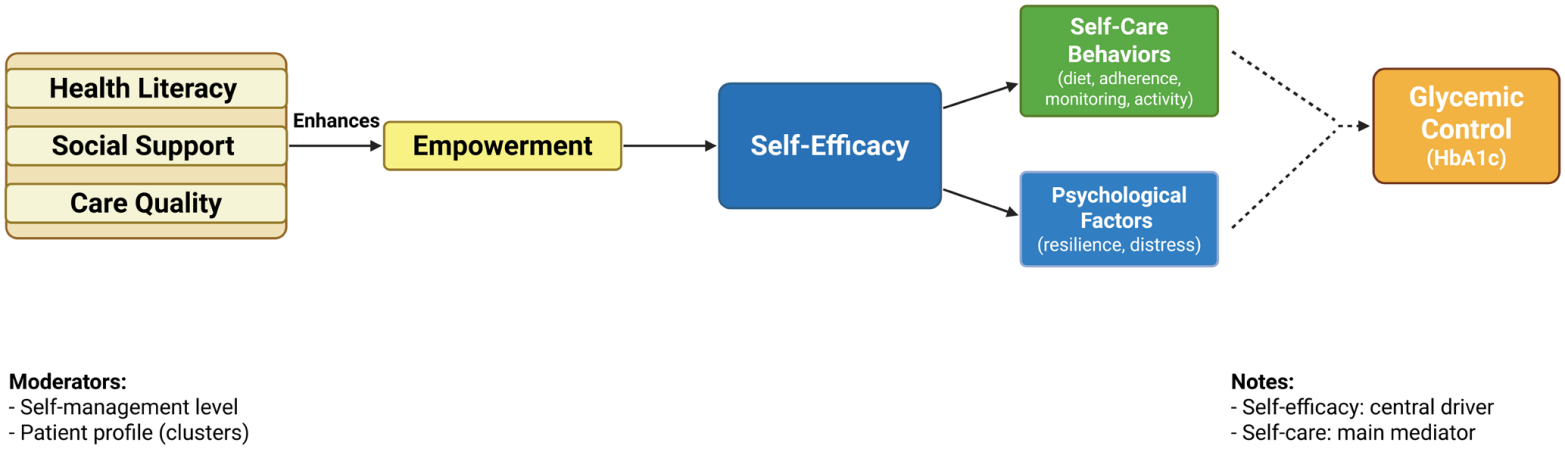
Conceptual framework of the relationship between patient empowerment, self‐efficacy and glycaemic control.

Figure 2 presents the integrated conceptual model synthesised from the included studies. The model identifies self‐efficacy as the primary determinant of self‐care behaviours, quality of life and glycaemic control. It also delineates the mediating roles of empowerment, self‐care and quality of life, as well as the moderating effects of self‐management levels and patient heterogeneity.

### 4.5 Clinical Heterogeneity and the Case for Stratified, Personalised Care

The cluster analysis by Nilmart et al. provides a methodologically distinct contribution by demonstrating that T2DM patients in routine care settings cannot be treated as a clinically homogeneous group [34]. The identification of four meaningfully different behavioural and psychological profiles, including the clinically significant paradox of the ‘underperforming’ cluster, in which high self‐efficacy coexists with poor self‐management and poor glycaemic control, challenges both one‐size‐fits‐all educational interventions and the assumption that self‐confidence is sufficient to drive behavioural change. This pattern, in which patients overestimate their self‐care capability relative to their actual performance, aligns with the broader psychological literature on miscalibration in self‐assessment and suggests that this cluster specifically requires structured, competency‐based skills training rather than motivational enhancement. The ‘High Risk’ cluster, comprising 10.2% of the sample and scoring lowest across all dimensions, represents a priority group for intensive, multimodal support. These findings align with the American Diabetes Association's guidelines for individualised diabetes management and support the integration of routine behavioural and psychological phenotyping into standard diabetes care workflows as a basis for tailored intervention planning [23].

### 4.6 Limitations

Several limitations of this scoping review must be acknowledged. First, in accordance with established scoping review methodology, no formal risk‐of‐bias or methodological quality appraisal was performed for the included studies; this decision limits the ability to weight findings by study quality and may affect the confidence with which interpretive conclusions are drawn. Second, no prospective protocol registration was completed before initiation of the review, which reduces methodological transparency. Third, the search was restricted to English‐language publications, potentially introducing language bias and excluding relevant evidence published in other languages. Fourth, the review was limited to four electronic databases (PubMed/MEDLINE, Scopus, ScienceDirect and CINAHL), and grey literature, including dissertations, conference proceedings, and government reports was not systematically searched, which may introduce publication bias. Fifth, the predominance of cross‐sectional designs among the included studies (*n* = 6 of 13) precludes causal inference regarding the directionality of associations between self‐efficacy, self‐care, glycaemic control and HRQoL. Sixth, heterogeneity in the instruments used to measure self‐efficacy (DMSE, SED, DMSES, GSE‐6), self‐care and HRQoL across studies limits direct cross‐study comparisons of effect sizes. Seventh, the geographic concentration of studies in East and Southeast Asian settings (eight of thirteen studies) restricts the generalisability of findings to other cultural and healthcare contexts, including African, Middle Eastern and Western populations.

## 5 Conclusion

Across all five thematic domains, the evidence synthesised in this scoping review converges on a coherent model of T2DM self‐management in which self‐efficacy appears to function as a central psychological factor associated with self‐care, though longitudinal evidence is needed to confirm directionality. Self‐care behaviours consistently mediate the relationship between care quality and glycaemic outcomes, while psychological constructs, such as empowerment, resilience and self‐management, modulate the strength of these associations across diverse populations. Theoretically grounded, individually tailored and explicitly self‐efficacy‐focused digital and structured educational interventions tend to show superior clinical effectiveness. The heterogeneity of patient profiles underscores the imperative for stratified, personalised approaches in diabetes care planning and intervention design.

## Author Contributions

**Omar Alqaisi:** conceptualization, methodology, software, data curation, investigation, writing – original draft, writing – review and editing, formal analysis, validation. **Faten Harb:** writing – original draft, writing – review and editing, software. **Safia Darwish:** writing – original draft, writing – review and editing, software. **Mohammed Dibas:** writing – review and editing, methodology, project administration. **Suzan Gharib:** writing – original draft, conceptualization. **Patricia Tai:** writing – review and editing, supervision.

## Funding

The authors have nothing to report.

## Consent

The authors have nothing to report.

## Conflicts of Interest

The authors declare no conflicts of interest.

## Supporting information


**Table S1:** Search String Strategy per Database.
**Table S2:** Scoping Review Check list.
**Table S3:** Studies Examining Self‐Efficacy as a Predictor or Mediator of Glycemic Outcomes.
**Table S4:** Summary of Digital Interventions and Comparator Self‐Efficacy‐Based Education Studies (*n* = 5 studies).

## Data Availability

Data sharing not applicable to this article as no datasets were generated or analysed during the current study.
