# Supplementary material for: Self‐Efficacy, Self‐Care, Glycaemic Control, and Quality of Life Among Adults With Type 2 Diabetes: A Scoping Review
Source: Endocrinol Diabetes Metab. 2026 May 20;9(3):e70241. doi: 10.1002/edm2.70241 (PMC13240330; doi:10.1002/edm2.70241)
Supplement: Supplementary file 1 — Table S1: Search String Strategy per Database. Table S2: Scoping Review Check list. Table S3: Studies Examining Self‐Efficacy as a Predictor or Mediator of Glycemic Outcomes. Table S4: Summary of Digital Interventions and Comparator Self‐Efficacy‐Based Education Studies (n = 5 studies). [file EDM2-9-e70241-s001.docx]

**Table S1.** Search String Strategy per Database

| Database | Search String | Filters Applied | Results |
| --- | --- | --- | --- |
| PubMed | ("diabetes mellitus, type 2"[MeSH] OR "type 2 diabetes"[tiab]) AND ("self-efficacy"[MeSH] OR "self-efficacy"[tiab] OR "self-management"[tiab] OR "diabetes self-management"[tiab]) AND ("glycemic control"[tiab] OR "HbA1c"[tiab] OR "hemoglobin A1c"[tiab] OR "quality of life"[MeSH] OR "digital health"[tiab] OR "mHealth"[tiab] OR "smartphone"[tiab]) | 2016–2026; English | 620 |
| Scopus | TITLE-ABS-KEY ("type 2 diabetes" AND ("self-efficacy" OR "self-management" OR "self-care") AND ("glycemic control" OR "HbA1c" OR "quality of life" OR "digital health" OR "empowerment")) AND PUBYEAR > 2015 | 2016–2026; English | 540 |
| ScienceDirect | "Type 2 diabetes" AND ("self-efficacy" OR "diabetes self-management") AND ("HbA1c" OR "glycemic control" OR "quality of life" OR "mobile health" OR "digital intervention") | 2016–2026; English | 480 |
| CINAHL | (MH "Diabetes Mellitus, Type 2") AND (MH "Self-Efficacy" OR TI "self-management" OR TI "self-care") AND (TI "glycemic control" OR TI "HbA1c" OR TI "quality of life" OR TI "digital health") | 2016–2026; English | 310 |
| Total |  |  | 1,950 |

**Table S2.** Scoping Review Check list

| **SECTION** | **ITEM** | **PRISMA-ScR CHECKLIST ITEM** | **REPORTED ON PAGE #** |
| --- | --- | --- | --- |
| **TITLE** | | | |
| Title | 1 | Identify the report as a scoping review. | P1 |
| **ABSTRACT** | | | |
| Structured summary | 2 | Provide a structured summary that includes (as applicable): background, objectives, eligibility criteria, sources of evidence, charting methods, results, and conclusions that relate to the review questions and objectives. | P1 |
| **INTRODUCTION** | | | |
| Rationale | 3 | Describe the rationale for the review in the context of what is already known. Explain why the review questions/objectives lend themselves to a scoping review approach. | P2-3 |
| Objectives | 4 | Provide an explicit statement of the questions and objectives being addressed with reference to their key elements (e.g., population or participants, concepts, and context) or other relevant key elements used to conceptualize the review questions and/or objectives. | P3 |
| **METHODS** | | | |
| Protocol and registration | 5 | Indicate whether a review protocol exists; state if and where it can be accessed (e.g., a Web address); and if available, provide registration information, including the registration number. | P3, protocol not registered |
| Eligibility criteria | 6 | Specify characteristics of the sources of evidence used as eligibility criteria (e.g., years considered, language, and publication status), and provide a rationale. | P4 |
| Information sources* | 7 | Describe all information sources in the search (e.g., databases with dates of coverage and contact with authors to identify additional sources), as well as the date the most recent search was executed. | P4 |
| Search | 8 | Present the full electronic search strategy for at least 1 database, including any limits used, such that it could be repeated. | P4 |
| Selection of sources of evidence† | 9 | State the process for selecting sources of evidence (i.e., screening and eligibility) included in the scoping review. | P4 |
| Data charting process‡ | 10 | Describe the methods of charting data from the included sources of evidence (e.g., calibrated forms or forms that have been tested by the team before their use, and whether data charting was done independently or in duplicate) and any processes for obtaining and confirming data from investigators. | P4-5 |
| Data items | 11 | List and define all variables for which data were sought and any assumptions and simplifications made. | Table 1 |
| Critical appraisal of individual sources of evidence§ | 12 | If done, provide a rationale for conducting a critical appraisal of included sources of evidence; describe the methods used and how this information was used in any data synthesis (if appropriate). | Not done, not mandatory |
| Synthesis of results | 13 | Describe the methods of handling and summarizing the data that were charted. | P8 |
| **RESULTS** | | | |
| Selection of sources of evidence | 14 | Give numbers of sources of evidence screened, assessed for eligibility, and included in the review, with reasons for exclusions at each stage, ideally using a flow diagram. | Fig. 1 on P5. |
| Characteristics of sources of evidence | 15 | For each source of evidence, present characteristics for which data were charted and provide the citations. | P8 |
| Critical appraisal within sources of evidence | 16 | If done, present data on critical appraisal of included sources of evidence (see item 12). | Not done, not mandatory |
| Results of individual sources of evidence | 17 | For each included source of evidence, present the relevant data that were charted that relate to the review questions and objectives. | P8-13 |
| Synthesis of results | 18 | Summarize and/or present the charting results as they relate to the review questions and objectives. | P8-13 |
| **DISCUSSION** | | | |
| Summary of evidence | 19 | Summarize the main results (including an overview of concepts, themes, and types of evidence available), link to the review questions and objectives, and consider the relevance to key groups. | Tables 1-4 |
| Limitations | 20 | Discuss the limitations of the scoping review process. | P16-17 |
| Conclusions | 21 | Provide a general interpretation of the results with respect to the review questions and objectives, as well as potential implications and/or next steps. | P17 |
| **FUNDING** | | | |
| Funding | 22 | Describe sources of funding for the included sources of evidence, as well as sources of funding for the scoping review. Describe the role of the funders of the scoping review. | P17 |

**Table S3.** Studies Examining Self-Efficacy as a Predictor or Mediator of Glycemic Outcomes

| **Author (Year)** | **Self-Efficacy Measure** | **Comparator Variables** | **Key Finding** | **Statistical Result** |
| --- | --- | --- | --- | --- |
| Hurst et al. (2020)  [37] | DMSE scale | Diabetes self-management (DSM), Diabetes knowledge (DK) | DMSE was the strongest independent predictor of HbA1c control after mutual adjustment | OR = 2.67 (95% CI: 2.20–3.25, P < 0.001); DSM attenuated to OR = 1.11 (ns) after DMSE adjustment |
| Kaveh et al. (2022)  [39] | SCT self-efficacy construct | Pre-post comparison (intervention vs. control) | SCT-based program significantly improved all SCT constructs and HbA1c | Self-efficacy change: +7.16 ± 0.63 vs. +0.02 ± 0.51 (p < 0.001); HbA1c: 8.29% → 6.28% (P < 0.001) |
| Jiang et al. (2019) [43] | Self-Efficacy for Diabetes (SED) | Usual care (control group) | Self-efficacy-focused education improved HbA1c and self-management at 6 months | HbA1c adj. mean diff: −0.740 (P < 0.001); SED: +0.517 (P < 0.001) |
| Lyu et al. (2021)  [42] | Diabetes Management Self-Efficacy Scale | Treatment adherence | Self-efficacy mediated the intervention's effect on HbA1c and QoL | Indirect effect: 0.18 (P < 0.05); variance in HbA1c explained: 52.5% |
| Zhang et al. (2024)  [44] | DMSES | SDM control group | Self-efficacy improved significantly with SDM-based digital dietary intervention | Time × group interaction: F = 7.127, P < 0.001 |
| Ting et al. (2025)  [36] | GSE-6 | Resilience, Self-management | Self-efficacy had direct and indirect effects on QoL; resilience partially mediated 43.1% | Direct effect: path c' = −0.753 (P < 0.05); total indirect: −0.781 (P < 0.001) |

CI: confidence interval; DMSE: Diabetes Management Self-Efficacy; DMSES: Diabetes Management Self-Efficacy Scale; DSM: Diabetes self-management; GSE-6: General Self-Efficacy Scale; ns: not statistically significant; OR: odd’s ratio; SCT: Social Cognitive Theory; SED: Self-Efficacy for Diabetes

**Table S4.** Summary of Digital Interventions and Comparator Self-Efficacy-Based Education Studies (n = 5 studies)

| **Author (Year)** | **Intervention Type** | **Duration** | **HbA1c Change (Intervention vs. Control)** | **Self-Efficacy Effect** | **QoL Effect** |
| --- | --- | --- | --- | --- | --- |
| Dobson et al. (2018)  [41] | SMS4BG text-message program (individually tailored) | 9 months | −8.85 ± 14.84 vs. −3.96 ± 17.02 mmol/mol; adj. diff. = −4.23 (P = 0.007) | No significant difference | EQ-5D VAS: +4.38 (P = 0.03) |
| Jiang et al. (2022)  [40] | Nurse-led smartphone app (NSSMP) vs. nurse-led diabetic service (NDS) | 6 months | No significant between-group difference | No significant difference | No significant between-group difference |
| Lyu et al. (2021)  [42] | Nurse-led web-based transitional care program | 3 months | ΔHBA1c = −2.87 (P < 0.01) | Indirect effect: 0.18 (P < 0.05) | ΔQoL = +7.69 (P < 0.01) |
| Zhang et al. (2024)  [44] | SDM-informed digital dietary intervention | 3 months | 6.92 ± 1.03 vs. 7.58 ± 1.13; t = 3.298, P < 0.001 | Time × group: F = 7.127, P < 0.001 | Not reported separately |
| Jiang et al. (2019)  [43] | Self-efficacy-focused structured education (non-digital, for comparison) | 6 months | Adj. mean diff.: −0.740 (95% CI: −1.045 to −0.434, P < 0.001) | +0.517 (P < 0.001) | Not reported separately |

Adj. diff.: adjusted mean difference; EQ-5D: EuroQol 5-Dimension; NDS: Nurse-led Diabetic Service; NSSMP: Nurse-led Smartphone-based Self-Management Programmed; SMS4BG: text message-based diabetes self-management support program; SDM: Shared Decision-Making; VAS: visual analog scale; Δ: change
